# Adult and child and adolescent psychiatrists' experiences of transition in anorexia nervosa: a qualitative study

**DOI:** 10.1186/s40337-022-00610-0

**Published:** 2022-07-04

**Authors:** Antoine Stocker, Lucie Rosenthal, Laure Mesquida, Jean-Philippe Raynaud, Alexis Revet

**Affiliations:** 1 Service Universitaire de Psychiatrie de l’Enfant et de l’Adolescent, CHU de Toulouse, Hôpital Purpan, Place du Dr Baylac, TSA 40031, 31059 Toulouse cedex 9, France; 2 Fédération Régionale de Recherche en Psychiatrie Et Santé Mentale Occitanie, FERREPSY Occitanie, 31000 Toulouse, France; 3 CERPOP, Université de Toulouse, Inserm, UPS, Toulouse, France

**Keywords:** Adolescent, Young adult, Anorexia nervosa, Transition to adult care, Psychiatrists, Mental health services, Qualitative research

## Abstract

**Background:**

Young patients suffering from anorexia nervosa (AN) frequently need further treatment in Adult Mental Health Services (AMHS). The transition period from Child and Adolescent Mental Health Services (CAMHS) to AMHS is a critical time, with a high risk of disengagement from healthcare. We explored physicians’ perspectives of the transition to triangulate the multiple perspectives of physicians, parents and those with a lived AN experience to more comprehensively characterize the challenges in this process of treatment transition.

**Methods:**

Using purposive sampling, we recruited 16 physicians confronted with transition in AN (adult psychiatrists, child and adolescent psychiatrists and pediatrician) and conducted semi-structured interviews, which were anonymized, transcribed, and analyzed following the reflexive thematic analysis framework.

**Results:**

Our analysis produced three main themes. First, a shared agreement on the transition’s malfunction, where participants depicted transition as a dissatisfying, violent event. Second, the conception of AN as a disorder with specific needs, challenging the transition process especially regarding physicians’ engagement. Finally, the ideal transition conceived as a serene experience of separation, with unanimous agreement on the necessity to start the transition depending on patients’ needs rather than their age, in order to turn transitions into moments of care.

**Conclusion:**

Our results are in line with other qualitative research studying transition in AN and in other chronic diseases, either focusing on the experience of healthcare workers, families, or patients. Our research shows transition in AN as an anxiety-inducing experience for physicians, patients and families alike. Moreover, we highlight a gap in the way physicians perceive and assist the patient’s greater autonomy, depending on their specialty. Helping physicians to manage their patient’s autonomy, which is a cornerstone of the transition readiness concept, could be a very efficient way to improve transitions in AN.

**Plain English summary:**

Anorexia Nervosa (AN) is a severe disease, which most of the time starts during adolescence. Transition from Child and Adolescent Mental Health Services to Adult Mental Health Services is at risk of disengagement from healthcare. In order to better understand this process, we interviewed expert physicians about their experiences of transition in AN using a qualitative thematic analysis which highlighted three main themes. First, a shared agreement on the transition’s malfunction. Second, the conception of AN as a disorder with specific needs challenging the transition process. Finally, the ideal transition conceived as a serene experience of separation, which needs to be started depending on patients’ needs rather than their age. We also show differences in the way physicians perceive and assist the patient’s greater autonomy acquired during the transition. Helping physicians to support their patients in acquiring autonomy, which is a cornerstone of the transition readiness concept, could be a very efficient way to improve transitions in AN.

**Supplementary Information:**

The online version contains supplementary material available at 10.1186/s40337-022-00610-0.

## Introduction

Anorexia nervosa (AN) is defined by the association of weight loss, fear of gaining weight and body image distortion [1]. This disorder has a severe prognosis, with a six times higher overall mortality rate compared to the general population [2]. The incidence is highest between ages 14 and 18, with an estimated lifetime prevalence rate of 1–2% [3, 4]. Although longer follow-up duration leads to higher remission rates, it is estimated that about half of the patients suffering from AN will experience a complete remission of the disease, whereas 20% will shift to chronicity with a persistence of the symptoms [5]. More severe symptoms, longer duration of the disorder and later onset during adolescence are associated with a worse prognosis and a higher use of mental health services in adulthood [6].

In a cohort of teenage patients with severe AN needing inpatient treatment, time to recovery was estimated between 57 and 79 months [7]. The natural course of adolescent-onset AN thus often leads to a necessary transition from Child and Adolescent Mental Health Services (CAMHS) to Adult Mental Health Services (AMHS). Transition is defined as “the purposeful, planned movement of adolescents and young adults with chronic physical and medical conditions from child-centered to adult-oriented health-care systems” [8]. Transition management is crucial to avoid treatment drop-out and disengagement from health services, at a time when the risk of developing emerging mental health problems is the highest [9]. However, differences in terms of professional culture and practices make transition a difficult process to handle for the physicians involved [10]. Transition-aged patients moreover face biological modifications and new social obligations that add to the complexity of the situation [9, 11].

Few specific interventions have been evaluated: a 2015 review [12] only found two specifically transition-focused programs, out of six studies that evaluated transition within mental-health services or programs. They both showed a promising impact on quality of life or adherence to ambulatory care, although negative aspects were overlooked by the authors. Qualitative studies included in this review reported multiple impediments in transitions but showed better results when using transition-dedicated meetings or personnel. All studies concerning existing mental health systems reported a lack of support and a difficulty for youth to access care. This is especially problematic in AN, where delay in access to care constitutes a negative prognostic factor [13]. Moreover, the efficacy of AN-focused ambulatory services proposed within a stepped-care model remains overall disappointing [14].

Qualitative research has scope to capture the nuances and complexity of the experience of transition from child to adult treatment services for AN. Many qualitative studies already provided insight on the matter and proposed hypotheses to improve patient’s healthcare by expanding our comprehension of the transition. However, they focused on patients, families, or healthcare teams but never exclusively on physicians involved in the process. They also reported concerns regarding their results’ generalizability, either due to limitations in the sampling process (participants coming from one specialized program only, or working specifically in inpatient services) or geographical context (most of the studies being conducted in the same country). In this context, we chose to conduct a qualitative study revolving around both child and adolescent psychiatrists and adult psychiatrists involved in the transition of patients suffering from AN. Our objective was to explore physicians’ perspectives of the transition to triangulate the multiple perspectives of physicians, parents and those with a lived AN experience to more comprehensively characterize the challenges in this process of treatment transition.

## Material and methods

We conducted a qualitative clinical study from February to September 2021, across two cities from south-western France.

### Sampling

We followed a purposeful sampling strategy, widely used in qualitative research, and selected participants according to their expertise on the research topic and the richness awaited from their contribution [19]. Thus, we contacted physicians who had a strong interest for AN, were recognized among their peers for their expertise in this field, or had an experience working in inpatient or outpatient services specialized in AN.

We reached out to a total of 27 practitioners either using phone calls or e-mails, regardless of their medical practice (inpatient, outpatient, private). Among the physicians we contacted, 16 were interviewed, nine did not respond and three refused to be interviewed: one because of a presumed lack of expertise on the subject, one because of a lack of time and one because he did not want his interview to be included in the analysis.

16 participants took part in the study (one third aged 30–40 years and 62.5% female). Our sample consisted of six child and adolescent psychiatrists and one pediatrician working exclusively with children and adolescents; two child and adolescent psychiatrists working or having worked with adolescents and adults; and seven psychiatrists working with adolescents and adults. The characteristics of the study participants are presented in Table 1.

**Table 1:** Characteristics of the study participants

N	Sex	Age	Professional background	Practice
1	F	30–40	Child and adolescent psychiatrist	Inpatient
2	M	30–40	Child and adolescent psychiatrist	Outpatient
3	M	30–40	Child and adolescent psychiatrist	Inpatient
4	F	40–50	Child and adolescent psychiatrist	Outpatient
5	M	> 60	Adult psychiatrist	Outpatient
6	F	30–40	Adult psychiatrist	In- and outpatient
7	F	30–40	Child and adolescent psychiatrist	Inpatient
8	F	40–50	Pediatrician	Inpatient
9	M	40–50	Child and adolescent psychiatrist	Inpatient
10	F	> 60	Adult psychiatrist	Private
11	F	40–50	Child and adolescent psychiatrist	Private
12	F	40–50	Child and adolescent psychiatrist	Private
13	F	20–30	Adult psychiatrist	In- and outpatient
14	M	50–60	Adult psychiatrist	In- and outpatient
15	M	> 60	Adult psychiatrist	Retired
16	F	30–40	Adult psychiatrist	Outpatient

### Data collection

Our data were composed of individual, semi-structured interviews, which were audio-recorded and transcribed in text form. Each participant was only met once, and every interview was conducted by AS, who systematically specified his position as a Child and Adolescent Psychiatry (CAP) trainee and his specific interest for this disorder. It is to note that AS knew several participants before the interviews, and already worked with three of them during his training. Assumptions coming from the interviewer were clarified as such during the interviews (e.g., the feeling that patients suffering of AN had a specific profile within healthcare and would benefit from dedicated transition interventions) to avoid influencing the interviewees’ point of view and prevent them from inhibiting their answers.

An interview (number 7) was conducted with two physicians (a child and adolescent psychiatrist and a pediatrician), at the request of the child psychiatrist, for whom it was important to enrich and put into perspective her experience with that of the pediatrician with whom she was accustomed to working and who was both specialized in clinical work with adolescents and in AN. Interviews lasted on average 45 min. Because of the safety precautions adopted during the coronavirus outbreak, six interviews were performed using videoconferencing and two over the phone. Two recent studies [20, 21] showed that qualitative research quality was not inferior when performing remote interviews compared to usual face-to-face interviews. Every face-to-face interview was conducted on the participant’s workplace.

The interview guide was written by AS and validated by two authors (LR and AR). Data gathering and analysis were pursued simultaneously, thus allowing us to include topics emerging from previous interviews in the interview guide. The final version of the guide is available as a supporting information file (see Additional file 1).

We chose to stop the analysis when the addition of a new interview did not modify our thematic map. This criterion is akin to the concepts of data saturation or thematic exhaustion, frequently used as a validity criterion [22] and sampling threshold in qualitative research [23]. It is however incoherent with the “organic”, constructivist approach of reflexive thematic analysis [24], where the researcher plays an active role in creating the themes during the research.

We delineated our three main themes after analyzing 10 interviews. We then analyzed four more interviews that provided new codes and enriched our main themes without altering them. The analysis of one final interview yielded no new themes nor subthemes.

Our final dataset consisted of 15 interviews conducted with 16 practitioners. Interviews were encoded by AS using the NVivo 12 software developed by QSR international.

Our study’s report fulfils the COnsolidated criteria for REporting Qualitative research (COREQ) Checklist, which proposes criteria to ensure the quality of qualitative research reporting [25].

### Data confidentiality and ethical aspects

This study was registered by the Toulouse University Hospital and the French Data Protection Authority (CNIL), which provided a reference methodology to ensure legal requirements regarding personal data were respected [26]. Audio recordings were destroyed after transcription. The anonymization process was an important point of our research: several participants directly raised their concerns regarding their anonymity, and it is possible that some of them inhibited their answers to avoid getting recognized by participants sharing their professional expertise [27]. We managed this issue of “internal confidentiality” by proceeding to an anonymization consistent with the context from which the data were extracted, thus preserving participants’ confidentiality without compromising data integrity [28]. Quotes illustrating our findings were individually validated with the participants whose interviews were used to produce the quotes, to remove any potentially identifiable information.

### Reflexive thematic analysis framework

We used the reflexive thematic analysis method, which aims to identify, analyze, organize, describe, and report themes highlighted among collected data to reach a better understanding of a complex phenomenon [29, 30]. This method is built around several steps, with the possibility to go back and forth between each step. Every step has to be documented and explained so that the reader can attest the trustworthiness of the research [31]. The steps are defined as follows:

1. Becoming familiar with every aspect of the data and start noticing significant patterns. We first meticulously transcribed each audio recording and read the transcripts multiple times. We aligned with the constructivist grounding of reflexive thematic analysis and chose a data-driven approach. Thus, we prevented from using theoretical assumptions by annotating transcripts after a first complete reading to avoid jumping to premature conclusions, and by keeping track of our own reflections during each reading to better stay focused on the interviewee’s perspective.
2. Generating as many codes (i.e., units of meaning related to the studied phenomenon) as necessary while processing through the whole dataset. We chose a systematic approach and coded the entirety of an interview’s verbatim before moving to the next one. We chose to keep our codes close to the surface meaning of the participants’ sayings. We kept a data-driven approach and did not use our theoretical knowledge on the matter, nor our preliminary findings, to pre-build codes and apply them on subsequent interviews.
3. Articulating these codes into meaningful themes depending on their complementarity, antagonism, similarity, or subsidiarity. Themes encompass the codes and form the basic units of the analysis. We started generating themes after encoding the first 10 interviews. We tested various associations, either based on the tension we perceived between the codes, or on our own reflections about the data that we kept in a research journal. Themes were constructed from our codes in an inductive way, but we allowed ourselves to formulate them in a way that would provide a deeper, more implicit level of meaning.
4. Reviewing the themes to ensure their consistency regarding the codes they contain as well as the whole dataset. We reviewed our candidate themes, confronting them to the whole dataset. Using investigator triangulation with two authors (AS, LR) during a dedicated session, we outlined a first thematic map of our data.
5. Naming themes to reflect the storytelling of the data they contain regarding the research subject. As our analysis gained in complexity, we defined sub-themes to better grasp different aspects of our data.
6. Formatting the research report to give a compelling answer to the research subject. We went through an iterative process of rereading each theme’s data to ensure our writing covered as much of their richness as possible. The final analysis was then proofread by the co-authors.

## Results

### Results of the reflexive thematic analysis

Our analysis produced three main themes, each having three to four subthemes. The first theme addresses factors perceived as hindrances in the transition process. The second theme discusses the specificities of AN and the specific needs of the patients who suffer from it. Finally, the third theme regroups the objectives of an ideal, well-managed transition, and proposes leads to achieve such a transition. A detailed list of all sub-themes, along with the number of references across the dataset for each theme, is presented in the Additional file 2.

### First theme: Adult or child and adolescent psychiatrists: a shared agreement on the transition’s malfunction

#### Difficulties referring to specialized services

Every practitioner we interviewed but two regretted insufficient resources to organize satisfying referrals, whether because of a lack of time, financial means, or availability of services. Moreover, adult and child and adolescent psychiatrists equally stressed how unclear limitations of each service’s scope turned transitions into tedious processes. Adult psychiatrists also reported insufficient training regarding AN. Only one participant (Interview no. 6) stated that transitions of patients suffering from AN were not more difficult than other transitions. These challenges provided one participant a strong feeling of helplessness when organizing transitions, as stated below.

“We are led, in fact, to receive teenagers and grown-ups, by lack of downstream services. Thus, since the transition is not done there either, since we are compensating, since we have the feeling that we are palliating for what doesn’t exist or doesn’t exist anymore well that’s good enough, and we don’t think too much about the transition.” (Interview no. 3)

**Fig. 1:**
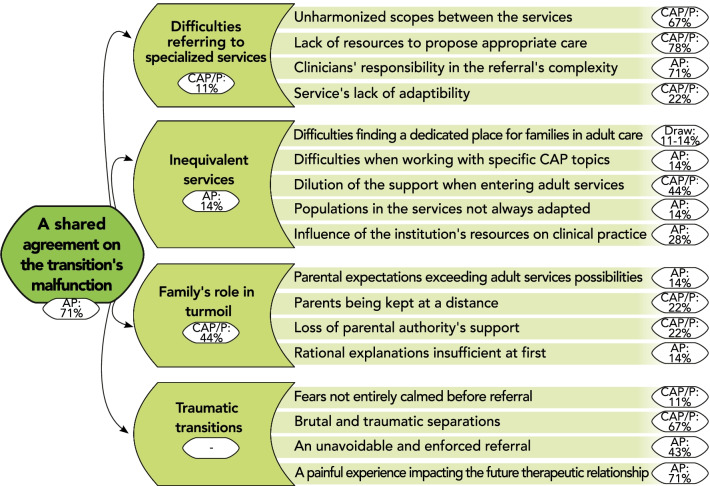
Thematic tree structure of the theme: “A shared agreement on the transition’s malfunction”. Themes and subthemes are presented with their coding rate among the subgroup in which they were the most encoded (proportion of participants expressing the theme within the subgroup, %). CAP/P: specialists in Child and Adolescent Psychiatry or Pediatrics; AP: specialists in Adult Psychiatry. Percentages from both CAP/P and AP subgroups are presented for equally represented themes (“Draw”). No rates are available (“–”) for overarching themes created without direct input from participants

Because of the persistent pressure under which they had to operate, clinical services did not prioritize transitions and lacked the flexibility to ensure satisfying referrals. The impossibility for practitioners to focus on transitions led to a perceived lack of availability of the interlocutors during referral, contrary to our participants’ belief in the necessity to take time when organizing transitions (Theme 3) (Fig. 1).

#### Inequivalent services

Most of the differences between AMHS and CAMHS were reported by adult psychiatrists only. They shared their concern that patient population in their services was not always adapted to patients suffering from AN and finishing their transition. Two pinpointed the differences in provision of care between services with a direct influence on proposed interventions. One reminded how hard it was to work on specific adolescent issues in an adult setting.

Adult and child and adolescent psychiatrists agreed on the lesser support to families proposed in adult services. They also agreed on the dilution of the support offered to the patient when entering adult services, which was stated by almost half of the child and adolescent psychiatrist and represented the most reported difference between services.

These differences were almost always perceived as troublesome restrictions by the practitioners. One participant expressed how painful it was for their patients to experiment such differing positions:

“And there is some sort of an all-or-none principle I think, here in the crossing of the healthcare pathway, between CAMHS which is, well, CAMHS, where we’re very close, very vigilant, patients are mentally supported by their doctor. And after that the adult transition where we’re more in a concern of, well in front of an adult. Yet they are not adults!” (Interview no. 10)

According to this participant, patients were not only subdued to considerable modifications of their usual medical care, but also underwent these changes in a very sudden, brutal way described within this theme (see below the “Traumatic transitions” subtheme). Practitioners were thus confronted with the difficulty of treating young patients as adults because of their age instead of their underlying maturity and autonomy (Theme 3).

#### Family’s role in turmoil

Adult care’s focus on the patient created a distance from the family which, according to our participants, was distressing for families as well as practitioners. Parents accustomed to CAMHS practices had high expectations, often exceeding adult services’ possibilities. These expectations raised, among adult physicians, the unpleasant feeling of having to justify themselves. Physicians were globally compassionate towards parents, although one retired adult psychiatrist remembered how difficult it was to reassure them (Interview no. 14). This anxiety arose not only from a lack of possibility to receive families, but also from a global change in care philosophy as stated in the following extract:

“For some it's quite... difficult, to... There are many who feel a little, who feel left out, who don't understand why we don't ask them, why we don't ask for their opinion, who will call several times to ask for appointments and who will get frustrated when we tell them, well, no, your daughter is of age, you can ask for her opinion.” (Interview no. 12)

When entering AMHS, patients frequently just reached majority and thus, were considered fully responsible of their medical healthcare. Parents were no longer consulted before medical decisions were made, and had to compromise with their child’s choice and the possibility of disagreeing with its outcome. This was exactly what worried almost half of the child and adolescent psychiatrists in our sample, who were afraid that patients would withdraw from care after reaching majority, when parental authority would no longer be something they could rely on.

#### Traumatic transitions

Transition was perceived as a brutal and inexorable event, experienced in a painful or even traumatic way by patients, families, and healthcare workers alike. Our participants strongly stressed how this feeling of distress left a negative impact on the working alliance. Half of the adult psychiatrists blamed the way transition was forced upon their patients and their services because of an age threshold, since patients turning 18 had to transition to AHMS regardless of the quality of their engagement in CAMHS. One of them questioned the appropriateness of some referrals, calling it “unfair” and “hazardous” (Interview no. 6). Age was not considered the only factor to bestow brutality on transitions in AN, as stated in the quote below.

“So anyway, there is often in anorexia, there can be anyhow the idea of not growing up and not separating from parents, and thus the adult referral is also caught in these stakes, uh, of incarnating the fact that their child grows and separates from them and thus there is often an already very violent experience of the adult services.” (Interview no. 1)

Transitions were thought to be even more traumatic for patients and their families because of the way AN challenges the familial relations beforehand. This participant perceived that the transition acted as an embodiment of a difficult reality caused by specificities of AN, in accordance with Theme 2. Moreover, two thirds of the child and adolescent psychiatrists we met dreaded the separation their patients would undergo, sometimes calling it “outrageous”.

### Second theme: Anorexia nervosa: a disorder apart

#### Clinical specificities hindering the transition process

Participants agreed on several specificities of these patients, mainly reported by child and adolescent psychiatrists, such as a lack of insight, difficulties in accepting the care framework or in projecting themselves into the future. This disease not only made transitions more arduous, but was also thought to spoil the adolescent process. Even though several participants argued that transition in AN does not fundamentally differ from transition in other disorders, others suggested caring for these patients constitutes a profession of its own. Organizing transitions in AN seemed to impose specific challenges on our participants, as emphasized in the following extract.

“Yes, I think transitions from minority to majority are a lot easier in other mental disorders. Because… Well actually it comes from the stiffness of anorexia, the severity of anorexia, that’s to say it is, well I think it is a very invasive disease, and we don’t have medications!” (Interview no. 11)

On top of the organizational and institutional challenges expressed by our participants in Theme 1, this practitioner felt that the severity of AN itself led physicians to deal with a strong feeling of helplessness. Other participants adopted this position, arguing that AN had a specific impact on the patient’s presentation, lessening the effectiveness of usual treatments and care frameworks.

#### An immature bonding calling for a strong commitment to care

Adult psychiatrists intensely stressed the difficulty of establishing and maintaining the therapeutic alliance, and were the only ones to mention the difficulty of engaging in these situations. On the other hand, child and adolescent psychiatrists especially felt that transitions paradoxically helped recreating momentum within the care. The tricky but necessary shutdown of the therapeutic relationship brought a welcomed change to situations perceived as static and immutable. The way practitioners were greatly affected when caring for patients suffering from AN was emphasized by one of our participants.

“It’s a slice of life you’re sharing, not a collection of symptoms to describe, not prescriptions to give even though you’re giving medications, it’s a slice of life you’re sharing. And that’s what’s going to leave a mark on them afterwards.” (Interview no. 14)

As highlighted in this quote, the way AN globally altered patients’ lives called for a strong investment of the physician in a holistic way, turning the therapeutic process as a whole into a lifetime event for the patients with a lasting effect on physicians themselves. Although bonding with patients suffering from AN could be challenging at first, patients often ended up strongly investing these relations, to the point of threatening the investment of a future healthcare team.

The transition moreover reactivated strong separation anxieties among the patients (see "Traumatic transitions" in Theme 1) and raised the question of a gap in terms of maturity upon arrival in the adult services.

#### Distinctive care arrangements matching the disorder's severity

Because of the strong impact of AN on family functioning, working with families was considered a cornerstone of AN treatment: participants with a CAP or pediatrics background unanimously agreed on this point, and insisted on proposing dedicated therapy to the parents, or directly helping them finding their place in adult services. The necessity of working hand in hand with other physicians to take care of somatic complications caused by AN was equally reported by adult and child and adolescent psychiatrists. The severity of AN symptoms altered the way physicians got in touch with their patient, as exemplified in the following extract.

“I don’t know if it’s specific to eating disorders but, in a kind of serious presentation of the disorder, with a malnutrition, a slowdown, all the symptoms we know actually, it’s true that we have a hard time discerning the life instinct, the emergence of the subject beyond the apparent rigidity of the disorder, which tends to smooth the individual a little I think.” (Interview no. 2)

**Fig. 2:**
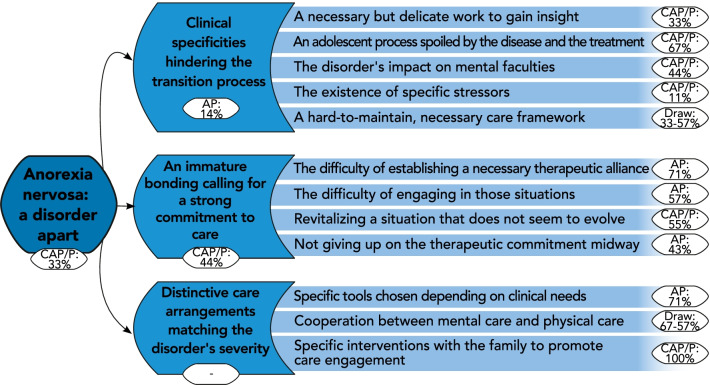
Thematic tree structure of the theme: “Anorexia Nervosa: a disorder apart”. Themes and subthemes are presented with their coding rate among the subgroup in which they were the most encoded (proportion of participants expressing the theme within the subgroup, %). CAP/P: specialists in Child and Adolescent Psychiatry or Pediatrics; AP: specialists in Adult Psychiatry. Percentages from both CAP/P and AP subgroups are presented for equally represented themes (“Draw”). No rates are available (“– “) for overarching themes created without direct input from participants

The “emergence of the subject”, however hard for physicians to grasp, was perceived as a necessary first step on which further therapeutic actions would be undertaken. AN characteristics preventing physicians from reaching the patient’s self imposed specific adaptations of the proposed care, such as using particular therapeutic approaches to facilitate the discussion and decenter the patient from weight considerations. Such specific clinical “tools”, including empowerment, were widely supported by adult psychiatrists (Fig. 2).

### Third theme: The ideal transition: a serene experience of separation

#### Soothing the transition to avoid burdening the patients with our anxieties

Transitions are marked with the uncertainty of what lies ahead. Knowing how severe the disease could be, some physicians, mostly working in child and adolescent psychiatry, expressed their difficulties letting go of their patients and their concerns about the transition possibly going wrong. The following extract emphasizes the distress physicians felt when facing transitions.

“At this moment I have a few patients who are almost 18 years old, I don't really know, I don't really know what will happen next. When child and adolescent psychiatry services will no longer be there for them. I'm like the parents, * laughs * I'm afraid of the 18 years mark and of the end of the follow-up in child and adolescent psychiatry.” (Interview no. 10)

This participant identified themselves with the parents, and dreaded the loss of control on future events that transition could impose, most notably when patients reached majority (see “Family’s role in turmoil” in Theme 1). Although they might sometimes feel as anxious as the patient’s own parents, child and adolescent psychiatrists were the only participants to explicitly state the need to tame these anxieties to promote a well-going transition. Physicians were fearful of the course of the disease, the possible legal implications of minors and adult patients being hospitalized in the same service, the physical condition of their patients, and their greater autonomy.

#### Using the transition as a moment of care

Transitions were thought to possibly positively influence future care either by supporting patients in resuming their follow-up, or by fostering future adhesion to care (the latter being expressed by every child and adolescent psychiatrists we met but one). Transition was indeed regarded as a special moment, where it might be easier to speak and where physicians could help the patient finding new supports and reference points for his or her forthcoming adult life. Two adult psychiatrists specifically expressed how transition management could help the patient facing future life changes. The conditions under which transitions could play a positive role in medical healthcare were better highlighted in the following quote.

“If we think about how to induce a process of becoming a subject, it becomes extremely effective. In other words, how do we provide ourselves with the means to work with the adolescent, so that he can take a more subjective stand, and so that the opportunity of the referral to AMHS marks this process?” (Interview no. 1)

The quoted participant stated how transitions only fully worked when they were tied to the clinical perception of the patient as a whole, grounded within their own theoretical background. Transitions could be used as a way to foster the patient’s self, which our participants believed was undermined by AN (Theme 2). Anticipating the transition, and helping patients go through their issues could thus instill meaning within the process and turned a simple referral into a therapeutic experience. Transition could then be perceived as an “opportunity” to support healthcare rather than a difficulty to overcome.

#### Taking the time to assist the transition, working together

This subtheme was very consensual among our participants: the necessity to ensure the continuity of the care was referenced through all the interviews but one. Physicians also largely stressed the need to take as much time as necessary to ensure the bonding of the patient to the downstream services, even if it meant overstepping the originally planned age limits. The quote below especially conveys the position our participants adopted regarding the timing of transitions.

“But anyway, I think that the time dimension of the transition is important, it’s not about meeting everyone at once one time and say goodbye. I think you have to weave things and unweave them afterwards, weaving with the adult side and progressively, like, let all the history of the ties to child and adolescent psychiatry go.” (Interview no. 3)

Transition was not defined as a single discrete event, but was rather perceived as a very time-consuming continuous process, a resource most of the services lack of (Theme 1). Interestingly, taking time when parting ways with old services was considered as important as taking time when meeting new ones, reflecting participants rejection of brutal transitions (Theme 1). Despite the constraints on the services, an abrupt breakup of the therapeutic relationship, leading to feelings of abandonment or care withdrawal, would be unacceptable.

Physicians emphasized the importance of knowing well enough the receiving services and professionals to properly support the patient undergoing transition. An idea widely supported by participants was thus to improve networking between AN specialists. To achieve this goal, adult psychiatrists especially insisted on the knowledge of AN care network, and the delineation of everyone’s practice to better identify other practitioners.

#### A transition matching clinical needs: supporting the patient’s autonomy

Age threshold imposed upon services did not make sense in clinical care to the majority of the interviewed physicians, who argued that these practices failed to take into account the patient’s needs, nor the reality of clinical practice. The physicians struggled against these stringent limitations, and believed their position was representative of patients and their families. All participants unanimously argued that the decision of referral should be tailored to suit the patient’s needs on a case-to-case basis and this position was the most encoded subtheme within our dataset and is exemplified in the following extract.

“We came to the idea (…) that we need to step out of this age-related thing, which is sometimes very rigid for everyone, to try and better adjust to the… personal issues of the individual, rather than a phenomenon we could globalize. And that maybe one of the keys to make it work would be some kind of individualized approach, and thinking the transition as a moment which can be a caring moment and not necessarily a painful moment” (Interview no. 6)

**Fig. 3:**
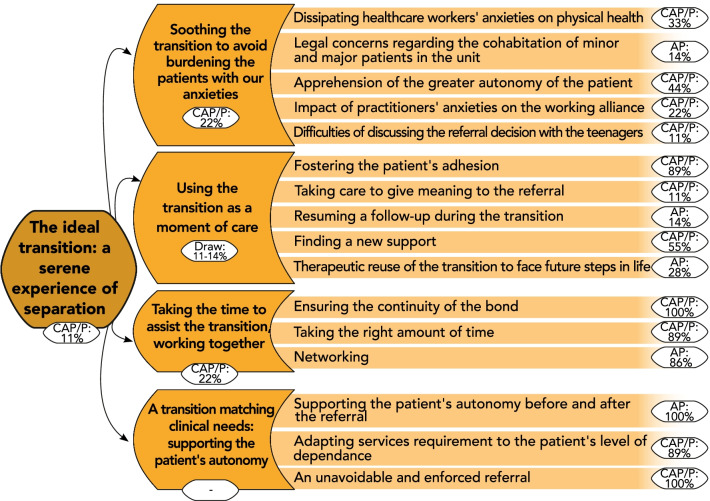
Thematic tree structure of the theme: “The ideal transition: a serene experience of separation”. Themes and subthemes are presented with their coding rate among the subgroup in which they were the most encoded (proportion of participants expressing the theme within the subgroup, %). CAP/P: specialists in Child and Adolescent Psychiatry or Pediatrics; AP: specialists in Adult Psychiatry. Percentages from both CAP/P and AP subgroups are presented for equally represented themes (“Draw”). No rates are available (“–”) for overarching themes created without direct input from participants

The perspective of this clinician was that personalizing transition to adult services, rather than globalizing based on the criteria of age, had scope to transform transitioning into adult services to an ethic of care rather than pre-existing, rigid rules that risked a traumatic transition (Theme 1). Rather than the individual’s age, practitioners wished to base their decisions on clinical criteria (symptomatic severity, diagnosis, psycho-emotional maturity level), and most notably on autonomy. Among our participants, the term “autonomy” regrouped various notions, such as independence, responsibility, and self-reliance. One adult psychiatrist perceived transition as an embodiment of the passage into adulthood, and AN as a “disease of growing up” (Interview no. 12). However, the participants knew that such a support was not always proposed to the patient due to constraints on the receiving services. While before the referral, the work on autonomy was mostly focused on gaining insight and facilitate engagement in care, it was more about learning to handle new stressors and avoiding care withdrawal after the referral (Fig. 3).

## Discussion

To our knowledge, this study is the first qualitative research on the representations of transition in AN focusing solely on the physicians involved on each side of the transition. Our analysis produced three main themes: a shared agreement on the transition’s malfunction; AN as a specific disorder with specific needs; the ideal transition as a serene experience of separation.

Our results are in line with those of similar qualitative studies conducted with healthcare professionals regarding transition in AN, whether it is about the disease and the impact of decreased parental involvement [15], the necessity to adapt to the patient’s needs rather than to a rigid age limit [16], or the difficulty of working together and of transferring the working alliance to future teams [17]. A recent qualitative study [32] using thematic analysis and focusing on general transition in mental health services proposed themes that were almost all similar to our findings. The only difference was the perceived lack of willingness from patients and families to get involved in adult care, which is the opposite of what our participants reported and could be due to the strong relationship between family and AN pointed out by our participants. The needs identified by our participants correspond to elements pinpointed as barriers to transition by patients and families in a recent qualitative study [18]: helping understanding the transition, anticipating the referral, involving the parents and the previous healthcare teams, increasing the support before entering adult services, etc.

Some themes are shared with qualitative research conducted with healthcare providers working on the transition in non-psychiatric chronic diseases. Families’ strong expectations towards adult care services, unsatisfying relationships with prior care providers, and the lack of resources are reported in a multi-specialty study among adult providers [33]. Concerns about the divergence in approaches, intensive care coordination and time requirements, or treatment nonadherence are found among adult endocrinologists handling the transition of patients suffering from type 1 diabetes [34]. It is interesting to note that in our study, differences in clinical approaches are not just regarded as a gap between services but are perceived by adult physicians as a positive feature. They indeed strongly reported how theoretical grounding could secure their practice and help patients and practitioners better navigate the health system and propose better referrals. The importance of identifying a practice suiting the patient and the family might be exacerbated in AN. Indeed, differences in representations of this disorder are thought to hinder the process of care [35].

Besides the themes already described in the literature, our research shows how painful and unsatisfying this experience is to the physicians, to the extent that one participant expressed his feeling of an “absence of transition” (Interview no. 3). Only one example of positive transition is reported across our data, while the others are mostly described with violent words (e.g., “trauma”, “brutality”, “shock”). There is an overarching continuity in our findings, as evidenced by the pervasiveness of anxiety across all of our main themes: whether it is a strongly perceived apprehension of adult referral, the need to show a very strong bond to reassure the patient’s fear of abandonment, or the fact that the ideal transition is imagined as an experience of transition stripped of everyone’s anxieties.

Our analysis also highlights the representation among physicians of AN as a specific disorder, possibly requiring special skills and interventions, or a different organization of care. Such specific interventions, dedicated to transition in AN have been described in the literature [36, 37] and are based on specific clinical needs also brought up by our participants. The Foundation for Research and Education in Eating Disorders (FREED) program for example revolves around family inclusion in the transition process, psychoeducation, and collaboration to imagine the care project. This program showed promising results, in particular a decreased weight loss before the start of treatment [38].

While physicians from both sides expressed similarities in their representations of the transition, their experiences of the patient’s greater autonomy acquired during transition were different. Subthemes such as “Apprehension of the greater autonomy of the patient” and other subthemes regarding the need to secure the patient (e.g., “Finding a new support”, “Ensuring the continuity of the bond”, “Taking the right amount of time”) are mostly supported by child and adolescent psychiatrists. Adult psychiatrists seem to be keener on supporting this newly acquired autonomy (the subtheme “Supporting the patient’s autonomy before and after the referral” being by far the most represented among adult psychiatrists in the whole dataset), including social aspects such as housing or finances. Autonomy is a cornerstone of the transition process and should be a priority target in potential transition interventions: factors related to patient’s autonomy, such as self-efficacy or responsibility in disease management are indeed significantly associated with transition readiness [39]. This is in line with the fact that autonomy level is perceived by our participants as the most adequate criterion to engage in transition. To our knowledge, no studies on transition readiness among patients suffering from AN have been conducted yet. Given the central role autonomy occupies in transition according to our participants, such a work is needed to better take care of these patients.

Our study presents several limitations. First, although our results are consistent with qualitative research in other countries and contexts, our sample was limited to two cities. However, this is the case with most qualitative research on the subject, where small and often monocentric samples, rather than being a limitation of the research and its generalization, are the strength of qualitative methods in being able to delve into the nuances and complexities of the participants' lived experiences. Similarly, while we chose to focus on physicians and therefore did not include representatives of other categories of caregivers, differences in age, gender and experience within our sample of physicians reinforced internal generalization. A meta-synthesis of the literature on this question might help overcoming local specificities and get a broader picture of transition in AN by including other stakeholders’ point of view. Second, we have already noted that the researcher conducting the interviews knew several of the participants prior to the interviews, and had already worked with three of them during his training. As Joseph A. Maxwell has pointed out [40], there are two major threats to the validity of qualitative studies: researcher bias, which is how the researcher's theories, beliefs, and perceptual lens influence his or her interpretation of the data, and the researcher's effects on the individuals being studied, often referred to as reactivity. In our study, both types of bias were potentially present, as the researcher's theories and perceptions may have been influenced and constructed by these previous collaborations, and, in the other direction, the responses of those he or she knew or had worked with may have been influenced by him or her. However, as Maxwell reminds us [40], the challenge of qualitative research is not to eliminate these biases (which is in fact impossible because that would mean eliminating the researcher's subjectivity), but to explain these possible biases and especially how they were anticipated and taken into account or minimized. We already stated that in our study, the researcher's assumptions were clarified as such during the interviews. Third, the written report of this research might lack of insight on our reflexive process for readers to properly understand the decisions we made during our thematic analysis, thus limiting our results’ trustworthiness [31]. Finally, our methodological choices when designing this study (e.g., focusing on specific professional backgrounds, conducting only individual semi-structured interviews, not having another researcher coding the data) limited the use of various triangulation methods (41). However, these choices were purposely made and are coherent with the epistemological position of reflexive thematic analysis.

## Conclusion

This qualitative study, addressing physicians’ representations of transition in AN, produced results in line with similar studies conducted among patients, families, or professionals. It also showed the important anxiety both felt and experienced by practitioners at various stages of this process. Having these elements in mind when designing specific interventions for transition could help physicians supporting the patient acquiring autonomy, thus strengthening transition readiness. Further studies are needed to support this hypothesis, especially since literature on transition readiness in AN is scarce.

## Supplementary Information

**Additional file 1.** Semi-structured interview guide. The latest version of the interview guide we used in our interviews, comprising eight questions.

**Additional file 2.** Detailed results of the thematic analysis. A complete and comprehensive table of our analysis’ results, showing coding rates for every subthemes.

## Data Availability

All data generated from our thematic analysis are either presented in the main text or in the provided tables, figures and additional files. Anonymized transcriptions of the interviews conducted during the study, in text form and in French, are available from the corresponding author, AS, upon reasonable request.

## Abbreviations

AN: Anorexia Nervosa
CAMHS: Child and Adolescent Mental Health Services
AMHS: Adult Mental Health Services
CAP: Child and Adolescent Psychiatry
COREQ: COnsolidated criteria for REporting of Qualitative research
CNIL: French Data Protection Authority
CAP/P: Specialists in Child and Adolescent Psychiatry or Pediatrics
AP: Specialists in Adult Psychiatry
FREED: Foundation for Research and Education in Eating Disorders
