# Supplementary material for: Adult and child and adolescent psychiatrists' experiences of transition in anorexia nervosa: a qualitative study
Source: J Eat Disord. 2022 Jul 4;10:92. doi: 10.1186/s40337-022-00610-0 (PMC9252565; doi:10.1186/s40337-022-00610-0)
Supplement: Supplementary file 1 — Additional file 1. Semi-structured interview guide. The latest version of the interview guide we used in our interviews, comprising eight questions. [file 40337_2022_610_MOESM1_ESM.docx]

**Additional file 1.** Semi-structured interview guide.

1. What representation do you have of the transition process?
2. Have you already faced specific challenges during these transitions?
3. What place do families occupy in the transition?
4. In a more comprehensive manner, which role does the transition play within the care of these patients?
5. What is the place of the age criterion according to you?
6. Do patients suffering from anorexia nervosa present a specific profile?
7. Does this condition have a specific impact on the transition process?
8. Which solutions could we imagine to improve this process?
