# Supplementary material for: Adult and child and adolescent psychiatrists' experiences of transition in anorexia nervosa: a qualitative study
Source: J Eat Disord. 2022 Jul 4;10:92. doi: 10.1186/s40337-022-00610-0 (PMC9252565; doi:10.1186/s40337-022-00610-0)
Supplement: Supplementary file 2 — Additional file 2. Detailed results of the thematic analysis. A complete and comprehensive table of our analysis’ results, showing coding rates for every subthemes. [file 40337_2022_610_MOESM2_ESM.docx]

**Additional file 2.** Detailed results of the thematic analysis.

| Main themes  Sub-themes ***• Emerging themes*** | Occurrences in interviews with specialists in child and adolescent psychiatry or pediatrics (% of participants within the subgroup) * | Occurrences in interviews with adult psychiatrists (% of participants within the subgroup) * |
| --- | --- | --- |
| **Adult or child and adolescent psychiatrist: a shared agreement on the transition’s malfunction** | 2 (11%) | 12 (71%) |
| Difficulties referring to specialized services | 1 (11%) |  |
| ***•*** *Unharmonized scopes between the services* | 7 (67%) | 6 (43%) |
| ***•*** *Lack of resources to propose appropriate care* | 20 (78%) | 18 (86%) |
| ***•*** *Service’s lack of adaptability* | 2 (22%) |  |
| ***• P****hysicians' responsibility in the referral’s complexity* | 8 (44%) | 15 (71%) |
| Inequivalent services | 5 (44%) | 6 (14%) |
| ***•*** *Difficulties finding a dedicated place for families in adult care* | 1 (11%) | 1 (14%) |
| ***•*** *Difficulties when working with specific child and adolescent psychiatry topics* |  | 3 (14%) |
| ***•*** *Dilution of the support when entering adult services* | 11 (44%) | 4 (43%) |
| ***•*** *Populations in the services not always adapted* |  | 4 (14%) |
| ***•*** *Influence of the institution's resources on clinical practice* |  | 6 (28%) |
| Family’s role in turmoil | 4 (44%) | 2 (28%) |
| ***•*** *Parental expectations exceeding adult services possibilities* |  | 4 (14%) |
| ***•*** *Parents being kept at a distance* | 8 (22%) | 5 (43%) |
| ***•*** *Loss of parental authority’s support* | 7 (44%) | 2 (28%) |
| ***•*** *Rational explanations insufficient at first* |  | 1 (14%) |
| Traumatic transitions |  |  |
| ***•*** *Fears not entirely calmed before referral* | 4 (11%) |  |
| ***•*** *Brutal and traumatic separations* | 8 (67%) | 3 (28%) |
| ***•*** *An unavoidable and enforced referral* | 2 (11%) | 8 (43%) |
| ***•*** *A painful experience impacting the future therapeutic relationship* | 6 (33%) | 17 (71%) |
|  |  |  |
| **Anorexia nervosa: a disorder apart** | 3 (33%) | 2 (14%) |
| Clinical specificities hindering the transition process |  | 2 (14%) |
| ***•*** *A necessary but delicate work to gain insight* | 14 (33%) | 8 (71%) |
| ***•*** *An adolescent process spoiled by the disease and the treatment* | 13 (67%) | 5 (43%) |
| ***•*** *The disorder's impact on mental faculties* | 4 (44%) | 3 (43%) |
| ***•*** *The existence of specific stressors* | 2 (11%) |  |
| ***•*** *A hard-to-maintain, necessary care framework* | 6 (33%) | 6 (57%) |
| An immature bonding calling for a strong commitment to care | 8 (44%) | 5 (28%) |
| ***•*** *The difficulty of establishing a necessary therapeutic alliance* | 7 (22%) | 10 (71%) |
| ***•*** *The difficulty of engaging in those situations* |  | 20 (57%) |
| ***•*** *Revitalizing a situation that does not seem to evolve* | 20 (55%) | 7 (57%) |
| ***•*** *Not giving up on the therapeutic commitment midway* | 2 (22%) | 9 (43%) |
| Distinctive care arrangements matching the disorder’s extent |  |  |
| ***•*** *Specific tools chosen depending on clinical needs* | 7 (33%) | 21 (71%) |
| ***•*** *Cooperation between mental care and physical care* | 15 (67%) | 15 (57%) |
| ***•*** *Specific interventions with the family to promote care engagement* | 50 (100%) | 18 (71%) |
|  |  |  |
| **The ideal transition: a serene experience of separation** | 1 (11%) |  |
| Soothing the transition to avoid burdening the patients with our anxieties | 2 (22%) | 1 (14%) |
| ***•*** *Dissipating healthcare workers' anxieties on physical health* | 5 (33%) | 1 (14%) |
| ***•*** *Legal concerns regarding the cohabitation of minor and major patients in the unit* |  | 1 (14%) |
| ***•*** *Apprehension of the greater autonomy of the patient* | 12 (44%) | 6 (43%) |
| ***•*** *Impact of practitioners' anxieties on the working alliance* | 5 (22%) |  |
| ***•*** *Difficulties of discussing the referral decision with the teenagers* | 2 (11%) |  |
| Using the transition as a moment of care | 1 (11%) | 1 (14%) |
| ***•*** *Fostering the patient's adhesion* | 18 (89%) | 4 (43%) |
| **•** *Taking care to give meaning to the referral* | 5 (11%) |  |
| ***•*** *Resuming a follow-up during the transition* |  | 1 (14%) |
| ***•*** *Finding a new support* | 10 (55%) | 1 (14%) |
| ***•*** *Therapeutic reuse of the transition to face future steps in life* |  | 2 (28%) |
| Taking the time to assist the transition, working together | 4 (22%) |  |
| ***•*** *Ensuring the continuity of the bond* | 36 (100%) | 16 (86%) |
| ***•*** *Taking the right amount of time* | 21 (89%) | 5 (43%) |
| ***•*** *Networking* | 12 (55%) | 29 (86%) |
| A transition matching clinical needs: supporting the patient’s autonomy |  |  |
| ***•*** *Supporting the patient’s autonomy before and after the referral* | 12 (44%) | 61 (100%) |
| ***•*** *Adapting services requirements to the patient's level of dependance* | 24 (89%) | 9 (71%) |
| ***•*** *A decision tailored to the needs of each situation* | 39 (100%) | 37 (100%) |

*Main themes and sub-themes values do not refer to total occurrences, but to the number of occurrences directly encoded within these themes.*
